# ‘Ear stones’ in crocodylians: a cross-species comparative and ontogenetic survey of otolith structures

**DOI:** 10.1098/rsos.211633

**Published:** 2022-03-23

**Authors:** Julia A. Schwab, Mark T. Young, Stig A. Walsh, Lawrence M. Witmer, Yanina Herrera, Zena L. Timmons, Ian B. Butler, Stephen L. Brusatte

**Affiliations:** ^1^ School of GeoSciences, Grant Institute, University of Edinburgh, James Hutton Road, The King's Buildings, Edinburgh EH9 3FE, UK; ^2^ National Museum of Scotland, Chambers Street, Edinburgh EH 1 1JF, UK; ^3^ Department of Biomedical Sciences, Heritage College of Osteopathic Medicine, Ohio University, Athens, OH 45701, USA; ^4^ CONICET. División Paleontología Vertebrados, Museo de La Plata, FCNyM, UNLP, La Plata, Argentina

**Keywords:** crocodylia, inner ear, ontogeny, otolith, vestibular system

## Abstract

The vestibular system of the inner ear is a crucial sensory organ, involved in the sensation of balance and equilibrium. It consists of three semicircular canals that sense angular rotations of the head and the vestibule that detects linear acceleration and gravity. The vestibule often contains structures, known as the otoliths or ‘ear stones’. Otoliths are present in many vertebrates and are particularly well known from the fossil record of fish, but surprisingly have not been described in detail in most tetrapods, living or extinct. Here, we present for the first time a survey of the otoliths of a broad sample of extant crocodylian species, based on computed tomography scans. We find that otoliths are present in numerous crocodylian species of different growth stages, and they continue to increase in size during ontogeny, with positive allometry compared to skull length. Our results confirm that otoliths are a common component of the crocodylian vestibular system, and suggest they play an important role in sensory detection. Otoliths are likely common, but overlooked, constituents of the inner ear in tetrapods, and a broader study of their size, shape and distribution promises insight into sensory abilities.

## 1. Introduction

The inner ear labyrinth forms a crucial part of the system of balance in vertebrates. The balance-related part of the labyrinth is composed of two distinct sensory structures: the three semicircular canals, which detect angular acceleration (rotations) of the head, and the utricle and saccule of the vestibule, which respond primarily to linear acceleration and gravity [1–3]. In many vertebrates, biomineralized structures are present in the saccule and/or utricle; these so-called ‘ear stones’ are known as otoliths or statoliths. These structures are composed of apatite (calcium phosphate) in cyclostomes and/or calcium carbonate (calcite, aragonite, vaterite) in gnathostomes [4,5]. Within Tetrapoda, otoliths are formed from aragonite in lissamphibians and calcite in birds and mammals, whereas the otoliths of living reptiles may be composed of either aragonite or calcite [4].

Developed in the otic capsule, these otolithic structures can appear in two forms: as monocrystalline ‘ear stones’ or as a polycrystalline otoconial mass, held together by an organic gel [4]. These structures include sensory hair cells with overlying calcium crystallites called otoconia that are embedded in a gel-like macula layer. Movements of the head result in mechanical stimulation of the hair cells as the otoconia move relative to the otolithic mass, and hence produce neuronal excitation that is processed within the vestibulocerebellum [6]. However, the saccule and utricle can also function as organs of hearing, for instance in fish [7].

Otoliths have been intensively studied in fish, as they are often found isolated in sediments after death and decomposition of the animal. Fish otoliths are often used for species identification and to help reconstruct the climate and temperature of palaeoenvironments (e.g. [8,9]). Far less is known, however, about the otoliths in other vertebrates, especially in tetrapods such as crocodylians. In 1882, Kuhn [10] mentioned one otolith that almost completely filled the saccule in the American alligator (*Alligator mississippiensis*), and in 1884, Retzius [11] described a large otolithic mass consisting of small otoconia that almost completely filled the saccule in the same species. Some recent studies on squamates note the presence of otoliths [12–14], but they were not described in detail, and the literature on comparative morphometry of otoliths in non-model tetrapods is generally lacking.

Here, we present for the first time data on the saccular otoliths of a broad sample of crocodylian species. Based on computed-tomography (CT) scans, we three-dimensionally reconstructed the endosseous space within the bony labyrinth and the otoliths contained therein. We report an apparently monocrystalline structure in the vestibule/saccule of the inner ear across modern crocodylians that continues to increase its size during growth, with hatchling/juvenile specimens generally having relatively smaller otoliths compared to subadults/adults. This represents a much-needed insight into a crucial sensory system that has not been described in tetrapods in detail, and could be identified in other CT-scanned extant and fossil specimens in the future, perhaps giving insight into species identification and diversity, palaeoenvironments and sensory abilities.

## 2. Material and method

### 2.1. Institutional abbreviations

FMNH, Field Museum of Natural History, Chicago, Illinois, USA; MNB, National Museum of the Bahamas, Nassau, The Bahamas; NMS, National Museum of Scotland, Edinburgh, UK; OUVC, Ohio University Vertebrate Collection, Athens, Ohio, USA; TCWC, Biodiversity Research and Teaching Collections, Department of Ecology and Conservation Biology, Texas, USA; TMM, Texas Memorial Museum, University of Texas, Austin, USA; UF, University of Florida, Florida Museum of Natural History, Gainesville, USA; USNM, National Museum of Natural History; Smithsonian Institution; Washington, USA; YPM, Yale Peabody Museum of Natural History, CT, USA.

### 2.2. Dataset

**Figure 1.**
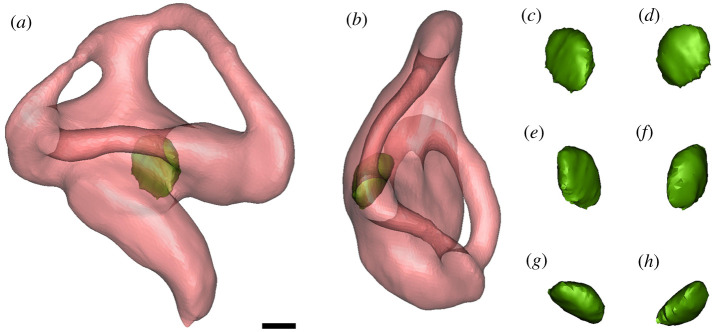
Transparent right endosseous labyrinth and otolith of *Melanosuchus niger* (NMS.Z.1859.13.804). Transparent right bony labyrinth in (*a*) lateral, (*b*) dorsal views; right otolith in (*c*) lateral, (*d*) medial, (*e*) posterior, (*f*) anterior, (*g*) ventral, (*h*) dorsal views. Scale bar equals 1 mm.

**Figure 2.**
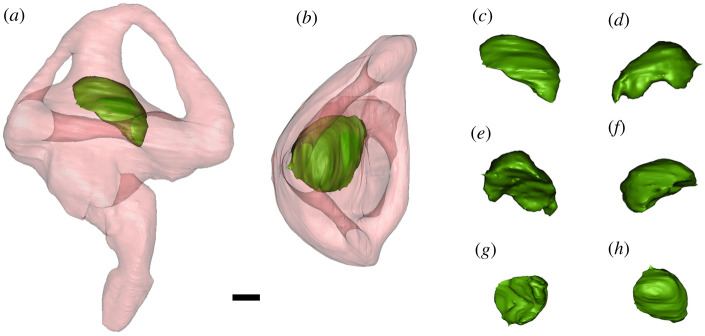
Transparent right bony labyrinth and otolith of *Osteolaemus tetraspis* (FMNH 98936). Transparent right bony labyrinth in (*a*) lateral, (*b*) dorsal views; right otolith in (*c*) lateral, (*d*) medial, (*e*) posterior, (*f*) anterior, (*g*) ventral, (*h*) dorsal views. Scale bar equals 1 mm.

**Figure 3.**
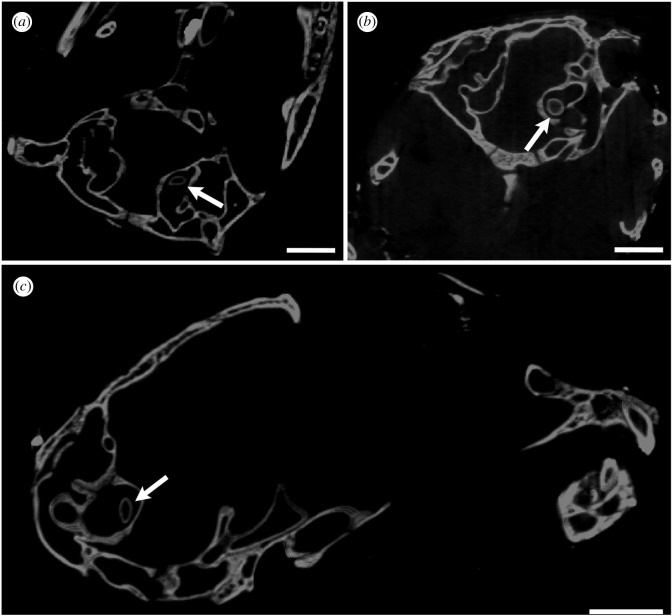
CT slices of *Melanosuchus niger* (NMS.Z.1859.13.804) with the otolith shown in the vestibule of the bony labyrinth (white arrow). (*a*) axial, (*b*) coronal, (*c*), sagittal views. Scale bars equal 5 mm.

**Figure 4.**
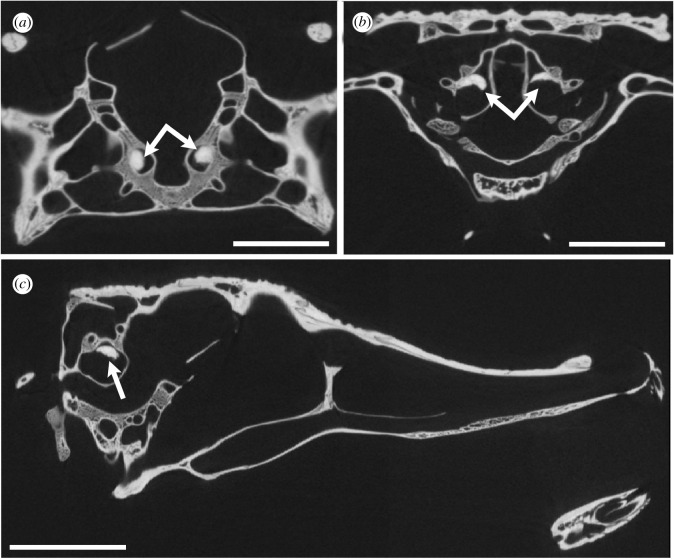
CT slices of *Osteolaemus tetraspis* (FMNH 98936) with the otolith shown in the vestibule of the bony labyrinth (white arrow). (*a*) axial, (*b*) coronal, (*c*), sagittal views. Scale bars equal 10 mm.

We analysed a total sample of 31 extant crocodylian bony labyrinths based on CT scans (for specimen details, table 1). Those crocodylian specimens include hatchlings, juveniles, subadults and adults, including members of the three major extant lineages (Alligatoridae, Crocodylidae and Gavialidae). Otolith-like structures were found in 20 of the specimens, but only 13 of those otoliths have clear boundaries that allow segmentation and were used for analysis (figures 1–4). Most specimens are dried skulls, however, this does not seem to have an impact on the presence of otoliths (table 1). There are multiple reasons why otoliths may not be preserved in all specimens. For instance, they may have been lost during preparation of the skull, or more likely, they may have broken up and are no longer apparent as a recognizable structure, while some otoliths were more polycrystalline and less cohesive and could have naturally disaggregated (as many of the specimens examined herein are osteological specimens). Otoliths were mostly found in both vestibules (with the exemption of two specimens).

**Table 1.** Detailed information on the specimens we included in this study.

specimen	ID	voxel size (mm)	otolith	ontogeny	dry/fleshy specimen
*Alligator mississippiensis*	OUVC 11415	0.0493	yes	juvenile	dried skull
*Alligator mississippiensis*	OUVC 10606	0.045	yes	hatchling	fleshy head
*Alligator mississippiensis*	UF-Herp-21461	0.0863526	no	juvenile	fleshy head
*Alligator mississippiensis*	TMM M-983	0.25 × 0.48	no	juvenile	dried skull
*Alligator mississippiensis*	USNM 211232	0.625	no	adult	dried skull
*Alligator mississippiensis*	USNM 211233	0.625	no	adult	dried skull
*Alligator mississippiensis*	OUVC 9761	0.5 × 1	yes	adult	dried skull
*Alligator mississippiensis*	NMS Unreg.	0.047145	right	juvenile	dried skull
*Alligator mississippiensis*	NMS Unreg.	0.04534	yes	hatchling	dried skull
*Alligator mississippiensis*	NMS Unreg.	0.031592	yes	hatchling	fleshy head
*Alligator sinensis*	TCWC 86174	0.512	yes	subadult	dried skull
*Alligator sinensis*	NMS Unreg.	0.084083	right	hatchling	fleshy head
*Caiman crocodilus*	FMNH 73711	0.065 × 0.142	yes	subadult	dried skull
*Crocodylus acutus*	FMNH 59071	0.625	no	adult	dried skull
*Crocodylus johnstoni*	TMM M-6807	0.223	no	subadult	dried skull
*Crocodylus moreletii*	TMM M-4980	0.1904 × 0.5	yes	adult	dried skull
*Crocodylus niloticus*	NMS.Z.1895.74	0.083602	yes	hatchling	fleshy head
*Crocodylus palustris*	NMS.Z.1968.13.55	0.101319	yes	juvenile	fleshy head
*Crocodylus porosus*	OUVC 10899	0.0472	yes	juvenile	fleshy head
*Crocodylus porosus*	NMS.Z.1925.9.1131	0.083602	yes	hatchling	fleshy head
*Crocodylus rhombifer*	MNB AB50.0171	0.1748 × 0.5	no	adult	dried skull
*Gavialis gangeticus*	UF-Herp-118998	0.14654672	no	adult	dried skull
*Gavialis gangeticus*	NMS Unreg.	0.053533	no	hatchling	fleshy head
*Gavialis gangeticus*	YPM HERR 008438	0.025390625	yes	hatchling	fleshy head
*Gavialis gangeticus*	TMM M-5490	0.228	no	subadult	dried skull
*Mecistops cataphractus*	TMM M-3529	0.165 × 0.5	yes	adult	dried skull
*Mecistops* sp.	NMS.Z.1859.13	0.078376	yes	hatchling	dried skull
*Melanosuchus niger*	NMS.Z.1859.13.804	0.083602	yes	hatchling	dried skull
*Osteolaemus tetraspis*	FMNH 98936	0.0546875 × 0.1108	yes	adult	dried skull
*Tomistoma schlegelii*	TMM M-6342	0.165 × 0.46	no	subadult	dried skull
*Tomistoma schlegelii*	USNM 211322	0.625	yes	adult	dried skull

**Figure 5.**
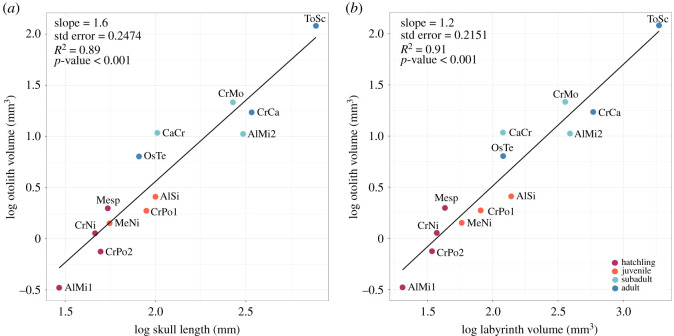
Plot of log-transformed (*a*) skull length (mm) and otolith mass (mm^3^), (*b*) labyrinth mass (mm^3^) and otolith mass (mm^3^) of crocodylians, indicating slight positive allometry. Colours indicate ontogenetic stages; magenta, hatchling; orange, juvenile; turquoise, subadult; blue, adult. Abbreviations are found in table 2.

Crocodylian skulls were scanned at various facilities, and scanning parameters vary (table 1). Three-dimensional models of the endosseous labyrinths and otoliths were created using Materialise Mimics 20.0, using the Livewire and Lasso tools. Only the right endosseous labyrinths were used as no significant left-right variation has been previously found [15,16]. The overall volume of the otoliths was taken as a size measure, and compared with the length of the skull (from the anterior point of the premaxilla to the occipital condyle) as a way to quantify otolith size (figure 5; table 2). We used the overall volume of the labyrinth as a size proxy, as there are no clear boundaries of the vestibule/saccule and other parts of the labyrinth to accurately segment out these structures. Those measurements were then log transformed and plotted against each other to test for allometry in RStudio [17].

**Table 2.** Measurements taken on the investigated specimens.

species	ID	Abbr.	skull length (mm)	otolith volume (mm^3^)	labyrinth volume (mm^3^)
*Alligator mississippiensis*	OUVC 10606	AlMi1	29.26	0.3321	20.37
*Alligator mississippiensis*	OUVC 9761	AlMi2	305.35	10.5894	389.7454
*Alligator sinensis*	NMS Unreg.	AlSi	99.85	2.5748	138.3306
*Crocodylus cataphractus*	TMM M-3529	CrCa	340.7	17.2028	586.8113
*Crocodylus moreletii*	TMM M-4980	CrMo	268.09	21.5928	358.5145
*Crocodylus niloticus*	NMS Z.1859.13.804	CrNi	46.24	1.132	37.2307
*Crocodylus porosus*	NMS Z.1925.9.1131	CrPo2	49.6	0.7487	34.2418
*Crocodylus porosus*	OUVC 10899	CrPo1	89.01	1.8765	80.7584
*Mecistops* sp.	NMS Z.1859.13	Mesp	54.39	1.9851	43.0245
*Melanosuchus niger*	NMS Z.1859.13.804	MeNi	55.69	1.419	57.7926
*Osteolaemus tetraspis*	FMNH 98936	OsTe	80.97	6.3736	119.9052
*Tomistoma schlegelii*	USNM 211322	ToSc	772.71	120.6766	1873.2885
*Caiman crocodylus*	FMNH 73711	CaCr	102.3	10.8348	119.5067

## 3. Results

**Figure 6.**
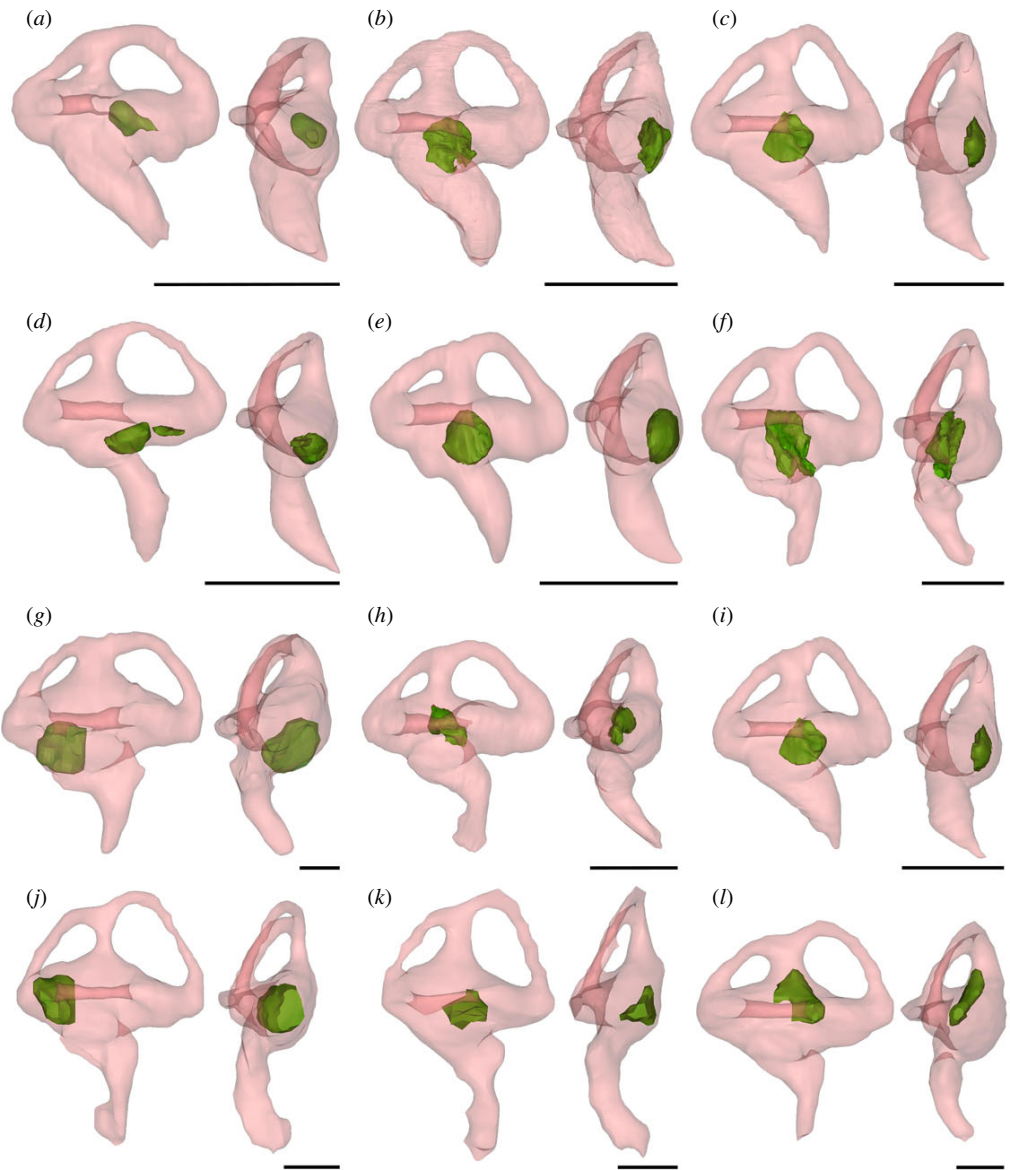
Right transparent endosseous labyrinths in lateral and dorsal views, with the otolith structure shown in green. (*a*) *Alligator mississippiensis* (OUVC 10606); (*b*) *Gavialis gangeticus* (YPM HERR 008438); (*c*) *Crocodylus porosus* (OUVC 10899); (*d*) *Crocodylus niloticus* (NMS.Z.1895.74); (*e*) *Mecistops* sp. (NMS.Z.1859.13); (*f*) *Caiman crocodilus* (FMNH 73711); (*g*) *Tomistoma schlegelii* (USNM 211322); (*h*) *Alligator sinensis* (NMS Unreg.); (*i*) *Crocodylus porosus* (NMS.Z.1925.9.1131); (*j*) *Crocodylus moreletii* (TMM M-4980); (*k*) *Alligator mississippiensis* (OUVC 9761); (*l*) *Mecistops cataphractus* (TMM M-3529). Scale bars equal 5 mm.

We observed a small otolith structure in the saccule of the inner ear in many modern crocodylians (figures 1,2, and 6). Based on our sample, we can confirm the presence of otoliths in alligatorids (*A. mississippiensis*, *A. sinensis* and *Caiman crocodilus*), crocodylids (*Crocodylus moreletii*, *Crocodylus niloticus*, *Crocodylus palustris*, *Crocodylus porosus*, *Mecistops cataphractus* and *Osteolaemus tetraspis*) and gavialids (*Gavialis gangeticus* and *Tomistoma schlegelii*) (table 1). Interestingly, the otoliths are mostly present either in both vestibules or neither. The otoliths in the examined crocodylian specimens occupy slightly different positions and are generally round-to-oval in shape. They are likely to have moved from an in-life position, as most of them are positioned on one side of the vestibule, contacting the wall of the labyrinth (figure 6). This is unsurprising, as the majority of our sample was composed of dry specimens, and hence the otoliths are likely to have diverted from their original position.

In the otolith-bearing specimens, smaller individuals generally have smaller otoliths (otolith volumes), with hatchling and juvenile specimens having smaller otoliths compared to subadults and adults. This observation is confirmed by our quantitative data, which demonstrates that the otolith structures grow with a slightly positive allometry in relation to both overall labyrinth volume and skull length (otolith volume versus skull length: slope = 1.66, *R*^2^ = 0.89, std error = 0.2474, *p*-value < 0.001; otolith volume versus labyrinth volume: slope = 1.2, *R*^2^ = 0.91, std error = 0.2151, *p*-value < 0.001). Two specimens, however, one subadult and one adult, do possess notably smaller otoliths compared to other specimens in their ontogenetic stage (figure 5). Those two specimens belong to *Caiman crocodylus* and *O. tetraspis*, which are two of the smaller sized crocodylians in the sample compared to the larger bodied specimens such as *A. mississippiensis*, *T. schlegelii*, *M. cataphractus* or *C. moreletii*.

The otolith structures are denser (as indicated by having higher Hounsfield units than the surrounding bone in the CT scans (visualized as the otoliths having brighter pixel values), which is consistent with X-ray attenuation of a material containing more calcium than hydroxyapatite [18]. In the hatchling and juvenile specimens, the otoliths appear to be hollow inside (figure 3). We are unsure whether the ‘hollow’ otoliths are a real ontogenetic feature or artefact of the adults being scanned at a lower resolution (due to their greater size).

## 4. Discussion

Otoliths were first mentioned in the American alligator in 1882 by Kuhn [10], and shortly after by Retzius [11]. However, since the 1880s there has not been a detailed examination of crocodylian otolith morphology, biochemistry, functional biology, or even a comparative survey of their presence among extant species. Herein we have taken the first step, and shown that not only are otoliths present in the American alligator vestibule throughout ontogeny but they are present in at least 11 species across all three extant families.

Given that otoliths are present across a range of extinct and extant vertebrates, it is not surprising that we find them in crocodylians. At the most fundamental level, our findings confirm that otoliths are a central component of the crocodylian inner ear sensory system, and constitute part of the neurosensory apparatus that crocodylians use to sense linear acceleration and gravity. Functionally, otoliths are involved in the sensation of linear acceleration, gravity and movements of the head. Might the size, shape, or position of the otoliths in crocodylians be linked to functional or behavioural traits? We here make a few observations and comparisons to place crocodylians in context with other tetrapods whose otoliths have been studied. We note there is a large otolith present in some burrowing squamates that can fill the entire saccular cavity [12,13]. This enlargement seen in squamates that burrow or rely on ground vibrations through the jaw (e.g. most snakes) might be an adaptation for low frequency vibration reception [19]. Unlike in squamates, the otoliths of crocodylians do not fill the entire vestibule, but instead are approximately one third of the size of the vestibule. This indicates that crocodylians and many squamates use some aspects of their inner ears differently to sense stimuli.

Interestingly, a particularly large vestibule compared to that of modern crocodylians has been noted in some of their fossil relatives: extinct Mesozoic marine and fully pelagic crocodylomorphs (the metriorhynchids), and the marine crocodyliform *Rhabdognathus* [16,20]. Unfortunately, no fossilized otolith structures have been discovered for fossil marine crocodylomorphs. If the otoliths in these fossil species filled the same proportion of the vestibule as in extant crocodylians, then the enlarged vestibules of metriorhynchids and *Rhabdognathus* would have contained otoliths larger than those seen in extant species. Relatively larger otoliths convergently evolved in different lineages of marine mammals, including seals, manatees and cetaceans [21], and hence there seems to be a link between larger otoliths and the marine environment in secondarily marine tetrapods, perhaps related to reduced gravitational effects in the water. In fish, this might be related to hearing capabilities, but also to balance and agility, as large pelagic species (tunas and swordfish) have relatively smaller otoliths compared to their body size, whereas shallow water reef fish have relatively larger otoliths [22]. We thus hypothesize that marine crocodylomorphs had larger otoliths within their enlarged vestibules and that changes to the vestibular-otolith system is yet another example of a sensory system that underwent transformation during the land-to-sea transition, along with the semicircular canals of the inner ear [16,23]. This remains to be tested by the discovery and description of fossil otoliths in these animals.

Another possibility is that differences in otolith size in crocodylians may be related to near-field hearing in water. McAngus Todd [24] suggested that the large, more fish-like otolithic sensor found in alligators might be specialized for particle motion detection, and is optimized for near-field reception of conspecific vocalizations under water. It is unclear whether captive/zoo animals have impacted otolith development, as these animals may not have used their senses in the same manner as a wild individual (e.g. different diet, unnatural habitat sustained close proximity to conspecifics), however the source of the specimen is not available for the majority of the specimens we studied.

The size of the otoliths in crocodylians, however, is generally correlated with the size of the animal, as well as their ontogenetic stage. Otoliths exhibit positive allometry—in other words, they get larger relative to ear and skull size in more mature individuals—suggesting that they are an important component of the sensory system, to which these individuals devoted mineral resources as they grew as larger otoliths can potentially host more sensory cells which would make them more sensitive. Hatchling and juvenile specimens generally have smaller otoliths compared to subadults and adults (figure 5) and if the otoliths simply maintained their size during ontogeny, or exhibited a proportional decrease in size, then their biological importance might seem to be less crucial. This is consistent with the ontogenetic signal found in the vestibular system of crocodylians where a significant size and shape change occurs during growth [25]. However, otoliths grow with a slight positive allometry compared to skull length and labyrinth volume, which contrasts with the size of the labyrinth, which grows with negative allometry in relation to skull length [25]. It is unclear whether such allometric growth would be consistent with optimization for hearing the vocalizations of conspecifics, and a broader sample of tetrapod otoliths is needed to determine whether there is anything remarkable about crocodylian otoliths (size or shape) that may be linked to their particular ways of vocalization.

Crocodylians, like many other tetrapod groups, lack the in-depth research into otoliths that has helped elucidate proprioception and equilibrioception in fish (e.g. [22]). More work is needed on otolith comparative anatomy across tetrapods, as well as research into otolith ontogenetic and evolutionary trends, and their biophysical and sensory functions. It is important to understand the link between the ‘ear stones’ and crocodylian hearing, balance and behaviour, but also how otoliths might have helped crocodylians adapt to an aquatic environment during their evolution.

## Data Availability

Three-dimensional labyrinth models have been uploaded to Morphosource (https://www.morphosource.org/) and can be accessed at https://www.morphosource.org/Detail/ProjectDetail/Show/project_id/952 and https://www.morphosource.org/projects/000384666.
